# Aging in the Right Place: A Conceptual Framework for Housing Insecure Older People

**DOI:** 10.1093/geroni/igab046.1128

**Published:** 2021-12-17

**Authors:** Rachel Weldrick, Sarah Canham, Atiya Mahmood, Tamara Sussman, Christine Walsh

**Affiliations:** 1 Simon Fraser University, Vancouver, British Columbia, Canada; 2 University of Utah, Salt Lake City, Utah, United States; 3 McGill University, Montreal, Quebec, Canada; 4 University of Calgary, Calgary, Alberta, Canada

## Abstract

Emerging research has highlighted the significance of aging in the right place (AIRP) by recognizing that secure and optimal housing should support an individual’s unique vulnerabilities and lifestyles. Existing literature, however, has yet to consider what it means for older people experiencing homelessness and/or housing insecurity to age-in-the-right-place. In order to address this knowledge gap, a review of person-environment fit models for older people and other relevant literature was conducted to determine critical identifiers of AIRP for housing insecure older people. Findings from this literature review were then refined in collaboration with interdisciplinary scholars and community partners to establish a conceptual framework. This paper presents the resulting conceptual framework and outlines the key indicators of AIRP relevant to housing insecure older people. The proposed framework provides a practical and meaningful contribution to the literature which can be used to promote housing security among individuals often excluded from existing aging-in-place models.

